# Cytokine Biomarkers of Exacerbations in Sputum From Patients With Chronic Obstructive Pulmonary Disease: A Prospective Cohort Study

**DOI:** 10.1093/infdis/jiae232

**Published:** 2024-06-05

**Authors:** Sonia Budroni, Marianna Taccone, Maria Stella, Susanna Aprea, Francesca Schiavetti, Monia Bardelli, Christophe Lambert, Simona Rondini, Vincent Weynants, Mario Contorni, Tom M A Wilkinson, Michela Brazzoli, Silvia Rossi Paccani

**Affiliations:** GSK, Siena, Italy; GSK, Siena, Italy; GSK, Siena, Italy; GSK, Siena, Italy; GSK, Siena, Italy; GSK, Siena, Italy; GSK, Wavre, Belgium; GSK, Siena, Italy; GSK Vaccines Institute for Global Health, Siena, Italy; GSK, Rixensart, Belgium; GSK, Siena, Italy; Clinical and Experimental Sciences, Faculty of Medicine, University of Southampton; National Institute for Health Research Southampton Biomedical Research Centre, Southampton Centre for Biomedical Research, Southampton General Hospital, Southampton, United Kingdom; GSK, Siena, Italy; GSK, Siena, Italy

**Keywords:** biomarkers, chronic obstructive pulmonary disease, cytokine signature, exacerbation, sputum

## Abstract

**Background:**

We determined the relationships between cytokine expression in sputum and clinical data to characterize and understand chronic obstructive pulmonary disease (COPD) exacerbations in people with COPD.

**Methods:**

We measured 30 cytokines in 936 sputum samples, collected at stable state and exacerbation visits from 99 participants in the Acute Exacerbation and Respiratory InfectionS in COPD (AERIS) study (ClinicalTrials.gov NCT01360398). We determined their longitudinal expression and examined differential expression based on disease status or exacerbation type.

**Results:**

Of the cytokines, 17 were suitable for analysis. As for disease states, in exacerbation sputum samples, interleukin (IL) 17A, tumor necrosis factor alpha (TNF-α), IL-1β, and IL-10 were significantly increased compared to stable state sputum samples, but a logistic mixed model could not predict disease state. As for exacerbation types, bacteria-associated exacerbations showed higher expression of IL-17A, TNF-α, IL-1β, and IL-1α. IL-1α, IL-1β, and TNF-α were identified as suitable biomarkers for bacteria-associated exacerbation. Bacteria-associated exacerbations also formed a cluster separate from other exacerbation types in principal component analysis.

**Conclusions:**

Measurement of cytokines in sputum from COPD patients could help identify bacteria-associated exacerbations based on increased concentrations of IL-1α, IL-1β, or TNF-α. This finding may provide a point-of-care assessment to distinguish a bacterial exacerbation of COPD from other exacerbation types.

## Teaser

Measuring 30 cytokines in 936 sputum samples from patients with chronic obstructive pulmonary disease, we observed significant increases (IL-17A, TNF-α, IL-1β, IL-10) between stable and exacerbation states. We also identified a combination of cytokines (IL-1α, IL-1β, or TNF-α) as suitable biomarkers for bacteria-associated exacerbations.

Chronic obstructive pulmonary disease (COPD) is a common, preventable, and treatable disease characterized by persistent respiratory symptoms associated with progressive airflow limitation, airway inflammation, and tissue damage [1]. COPD is accompanied by episodes of acute exacerbations (AEs), which are characterized by worsening symptoms, lead to faster lung function decline, and are a major cause of COPD-related morbidity and mortality [2, 3]. Airway inflammation during AE is characterized by heterogeneous secretion of inflammatory markers in the respiratory tract and serum. This heterogeneity is due to different exacerbation types [4, 5]. Based on inflammatory profiling, 4 exacerbation subgroups have been identified (bacterial [B], viral [V], or eosinophil [E] predominant, and pauci-inflammatory [Pauci]) [6] that may lead to optimal treatment if the exacerbation subgroup can be identified quickly.

To study AE of COPD (AECOPD) incidence and the pathogens involved, a longitudinal study, Acute Exacerbation and Respiratory InfectionS in COPD (AERIS), was conducted, in which COPD patients with a history of AE were followed monthly for 2 years [7]. The current exploratory study aimed to analyze the relationships between cytokine expression in sputum and patients’ clinical data to better understand COPD and distinguish between exacerbation types.

**Figure 1.**
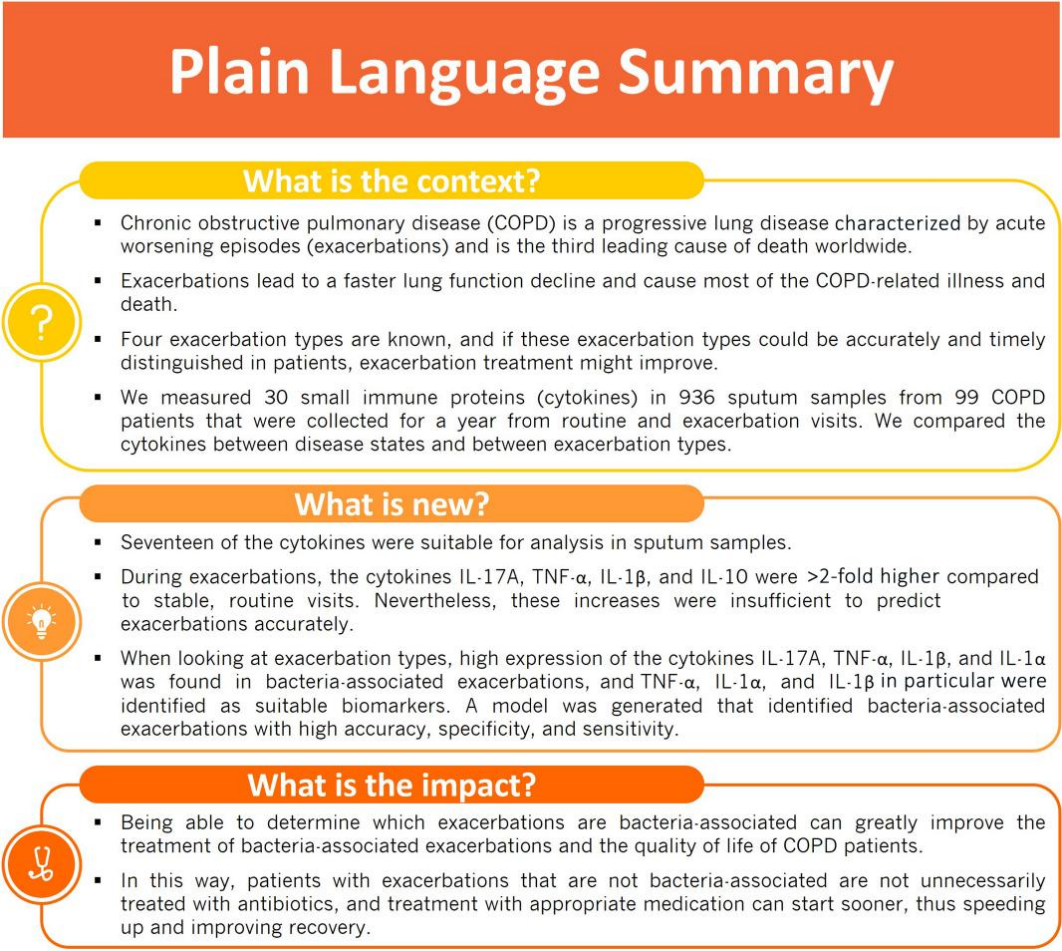
Plain language summary of the manuscript. Abbreviations: COPD, chronic obstructive pulmonary disease; IL, interleukin; TNF, tumor necrosis factor.

We investigated the lung cytokine profile in sputum samples from the AERIS study by quantifying 30 cytokines in sputum samples collected from 99 patients at stable state and exacerbation visits. Univariate and multivariate analyses addressed cytokine expression dynamics, comparing stable and exacerbation states to define clusters and cytokine signatures associated with viral, bacterial, and inflammation-associated exacerbations. Figure 1 summarizes this study’s context, outcomes, and the impact for healthcare professionals.

## METHODS

### Design and Setting

The AERIS study was a single-center, prospective, observational cohort study conducted at the University Hospital Southampton, United Kingdom, registered with number NCT01360398 (ClinicalTrials.gov). The AERIS study was conducted in accordance with the Declaration of Helsinki and Good Clinical Practice and has been approved by the institutional ethics and review committee. All participants provided written informed consent. COPD patients aged 40–85 years were included according to the AERIS study criteria [7]. Because patients were lost to follow-up or withdrew from the study mainly during the second year, we analyzed only samples collected during the first year to avoid possible bias due to missing outcome data. We selected participants with ≥3 available visits (with or without exacerbation).

### Sample Collection

All procedures for sputum sampling and pathogen detection were described previously [7, 8]. Sputum sample processing procedures for cytokine testing are available in the Supplementary Methods. In brief, sputum samples were collected at study entry, monthly, and ≤72 hours of exacerbation by spontaneous expectoration or induction and processed with standard methods. The visits were designated either stable state (ST) or exacerbation state (EX) based on disease state. EX represents the first exacerbation visit before treatment. Pathogens were identified by culture and polymerase chain reaction (PCR) [9].

### Exacerbation Definition and Types

In the AERIS study, exacerbation was defined as (1) worsening of 2 or more major symptoms (dyspnea, sputum volume or purulence), or (2) worsening of at least 1 major and 1 minor symptom (wheeze, sore throat, cold, cough, and fever) [7]. In addition, on exacerbation, patients were required to go to the clinical center for sample collection. Patient continued to visit the clinical center monthly for evaluation.

Samples collected before a patient's recovery from an exacerbation were excluded from the cytokine analysis as ongoing treatment may interfere with cytokine levels.

The samples’ exacerbation types were defined as:

- Bacterial (B) if DNA extracted from sputum was PCR positive for at least 1 of *Haemophilus influenzae*, *Streptococcus pneumoniae*, *Staphylococcus aureus*, *Moraxella catarrhalis*, or *Pseudomonas aeruginosa*.
- Viral (V) if DNA extracted from sputum was PCR positive for at least 1 of the viruses tested (Supplementary Table 1).
- Eosinophilic (E) if sputum contained >3% eosinophils of nonsquamous cells or if a blood sample contained >2% eosinophils of total leukocytes.
- Pauci if the sample was not defined as B, V, or E.
- Mixed if >1 type (B, V, or E) was detected. Mixed samples were excluded from the analysis.

### Measurements

We quantified 30 cytokines and chemokines (hereafter “cytokines”) with the Meso Scale Discovery (MSD; Meso Scale Diagnostics LLC, Rockville, Maryland) electrochemiluminescence V-plex human cytokine 30-plex panel kit (Supplementary Table 2) following manufacturer’s protocols. Plates were read by the MSD SECTOR Imager 6000 instrument, and data were analyzed using MSD Discovery Workbench software.

Assay characterization was performed to demonstrate that the method had adequate reproducibility for the intended purpose, establish plate and sample acceptance criteria, and confirm the quantification limits provided by MSD. Acceptance criteria as well as spike and recovery experiments are detailed in the Supplementary Methods. Precision analysis showed that the factors “day” and “operator” had no impact on assay variability and that the majority of cytokines showed coefficient variation <25%.

### Statistical Analyses

A linear mixed-effects model evaluated the differences between groups (ST vs EX) or exacerbation types (B, V, E, Pauci) for each cytokine. A logistic mixed model considered the contribution of all cytokines in predicting disease states simultaneously. A principal component analysis (PCA) identified the principal components (PCs) that accounted for the dataset variation. To identify biomarkers, the receiver operating characteristic (ROC) curve and area under the curve were plotted for bacteria-associated versus other EX and for bacteria-associated versus other ST.

## RESULTS

### Patients and Samples

We included 936 sputum samples from 99 participants (3–18 samples per participant, median: 10). Participants’ characteristics are in Table 1.

**Table 1.** Participant Characteristics

Characteristic	Participants (N = 99)
Age at enrollment, y, mean ± SD	66.9 ± 8.5
Female sex, No. (%)	43 (43.4)
COPD severity status at enrollment, No. (%)^a^	
Mild	0 (0.0)
Moderate	34 (46.6)
Severe	31 (42.5)
Very severe	8 (11.0)
No. of exacerbations during the 12 study months	
Mean ± SD (range)	2.4 ± 2 (0–8)
Median (IQR)	2 (1–3)

Abbreviations: COPD, chronic obstructive pulmonary disease; IQR, interquartile range; SD, standard deviation.

^a^Available for 73 patients.

### Cytokine Quantification

The 30 cytokines were measured in the 936 sputum samples (raw data in Supplementary Figure 1). For each cytokine, the values below the lower limit of quantification (LLOQ) were set to the arbitrary low value of LLOQ/4, and the values above the upper limit of quantification (ULOQ) were set to the arbitrary high value of ULOQ × 4. In subsequent analyses, cytokines were included only if ≥20% of the values were between the LLOQ and ULOQ. To avoid possible bias, cytokines for which ≥8% of samples did not generate valid results were excluded. This left 17 cytokines for analysis (Supplementary Table 2).

### Four Cytokines Were Increased in EX Compared to ST

**Figure 2.**
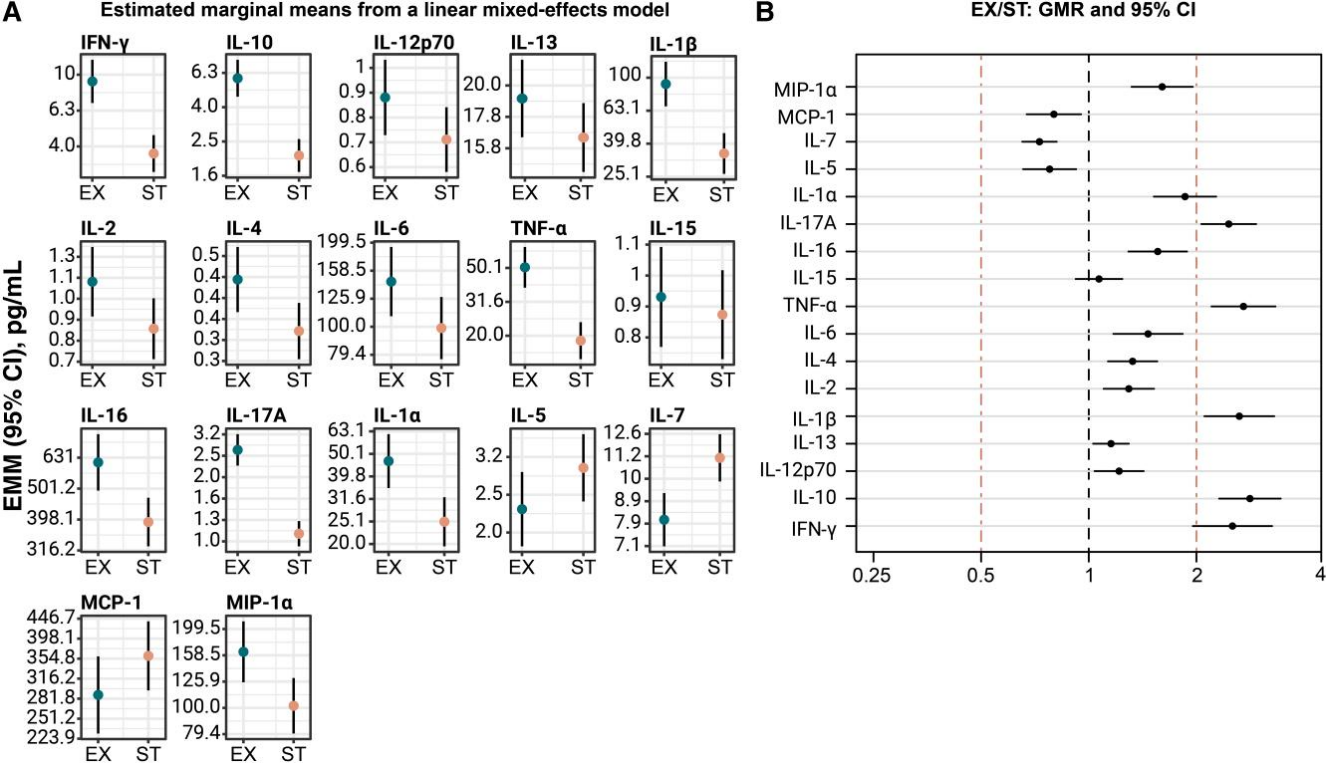
Sputum cytokines differentiate between stable state (ST) and exacerbation state (EX). Cytokine concentrations were measured in sputum samples (n = 936) obtained from 99 participants during ST (n = 701) and EX (n = 235) visits. *A*, Depicted are the estimated marginal means (EMMs) from a linear mixed-effects model. Vertical axis shows cytokine concentrations in pg/mL. Circles: EMM; bar: 95% confidence interval of the EMM. *B*, Geometric mean concentration (GMC) ratios of EX over ST. The GMCs at EX of IL-17A, TNF-α, IL-1β, and IL-10 are >2-fold higher compared to the GMCs at ST. Abbreviations: CI, confidence interval; EMM, estimated marginal mean; EX, exacerbation state; GMR, geometric mean ratio; IFN, interferon; IL, interleukin; MCP, monocyte chemoattractant protein; MIP, macrophage inflammatory protein; ST, stable state; TNF, tumor necrosis factor.

To identify differences in concentrations of the 17 cytokines between EX and ST, the estimated marginal mean and 95% confidence interval (CI) were computed for each cytokine using a linear mixed-effects model (Figure 2*A*). The geometric mean ratio (GMR) of EX over ST was then calculated for each cytokine. Univariate analysis showed that during EX, interleukin (IL) 17A, tumor necrosis factor alpha (TNF-α), IL-1β, and IL-10 concentrations were significantly increased (lower limit of the GMR 95% CI >2) compared to ST (Figure 2*B*).

A multivariate logistic mixed model was applied to consider simultaneously the contribution of all cytokines in predicting the disease state. The dataset was randomly split into a training (75% of data) and a test set (25% of data) to evaluate the model’s performance. The independent test set showed that the model had 88% accuracy and 97% specificity but only 39% sensitivity, which is insufficient to predict the disease state. Only IL-10 and IL-17A had a significant but weak positive effect (data not shown).

### Specific Cytokine Signatures Mark Exacerbation Types and Disease States

**Figure 3.**
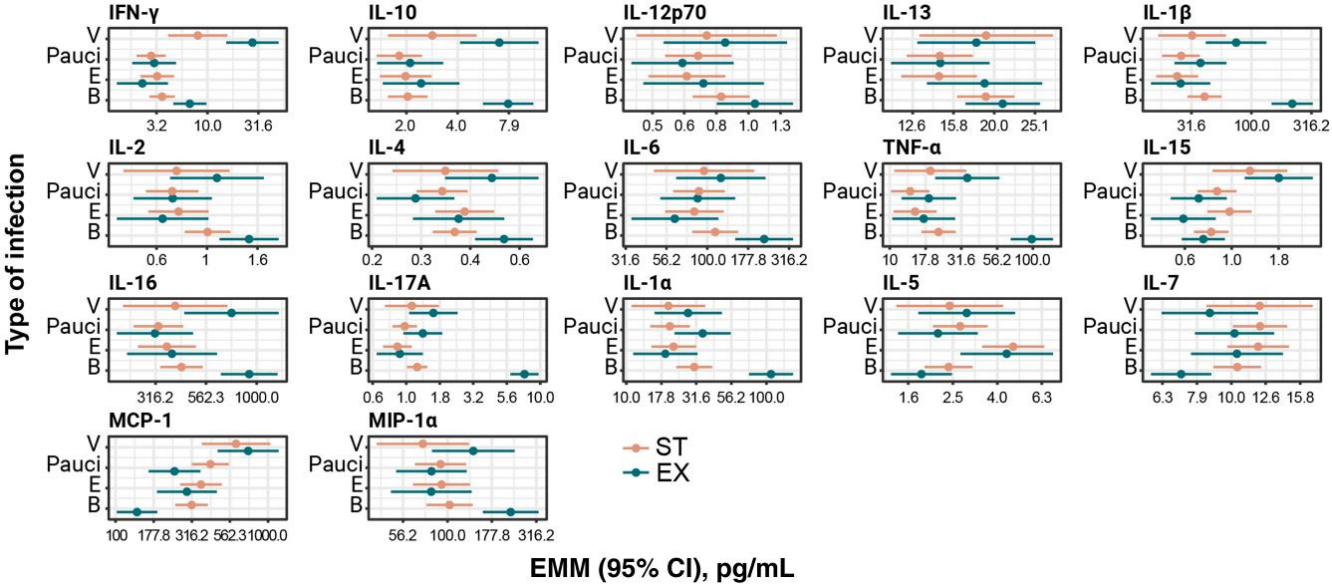
Sputum cytokine signatures correlate with the type of exacerbation (bacterial [B], eosinophilic [E], viral [V], Pauci) and disease state. Cytokine concentrations were measured in sputum samples (n = 513) obtained from 99 participants during stable state (ST; n = 369) and exacerbation state (EX; n = 144) visits (samples with mixed exacerbation types were not included). Depicted are the estimated marginal means (EMMs) and 95% confidence intervals (CIs) computed using a mixed effect specific for each cytokine grouped by type of exacerbation and disease state. Note that mixed exacerbation types (BV, BE, VE, BVE) were excluded, leaving 513 sputum samples for this analysis. Number of samples in each group: n = 16 in V ST, n = 106 in Pauci ST, n = 69 in E ST, n = 178 in B ST, n = 21 in V EX, n = 33 in Pauci EX, n = 22 in E EX, and n = 68 in B EX. Horizontal axis shows cytokine concentration in pg/mL. Circles: EMM; bar: 95% CI of the EMM. Abbreviations: B, bacterial; CI, confidence interval; E, eosinophilic; EMM, estimated marginal mean; EX, exacerbation state; IFN, interferon; IL, interleukin; MCP, monocyte chemoattractant protein; MIP, macrophage inflammatory protein; ST, stable state; TNF, tumor necrosis factor; V, viral.

When grouping samples by exacerbation types (B, V, E, or Pauci) and disease states (ST or EX), specific cytokine signatures emerged for the various groups (Figure 3). For instance, interferon gamma (IFN-γ) was high in EX samples with a viral pathogen, and IL-10 was high in EX samples with any pathogen (B or V).

### A Proinflammatory Cytokine Signature Correlates With Bacterial EX

A clear and significant separation between groups was observed for bacteria-associated exacerbations. The resulting proinflammatory cytokine signature was based on high expression of IL-17A, TNF-α, IL-1β, and IL-1α (Figure 3). In contrast, no cytokine signature could differentiate between the other exacerbation types.

To identify biomarkers for bacteria-associated exacerbations, ROC curves were analyzed in an ad hoc dataset obtained by randomly selecting 1 exacerbation per participant (63 samples) (Supplementary Figure 2). The most suitable biomarkers for determining bacteria-associated exacerbation were IL-1α (a threshold of 46 pg/mL had 80% sensitivity and 82% specificity), IL-1β (a threshold of 125 pg/mL had 66% sensitivity and 93% specificity), and TNF-α (a threshold of 40 pg/mL had 74% sensitivity and 89% specificity).

To investigate the combined contribution of IL-1α, IL-1β, and TNF-α as biomarkers for bacteria-associated exacerbations, we evaluated whether sensitivity and specificity changed if the levels of IL-1α, IL-1β, and TNF-α were all (or 2 of 3) above the respective thresholds. The biomarker combinations increased the specificity of predicting bacteria-associated exacerbations to 97%–100% (Supplementary Table 3).

### Bacteria-Associated Exacerbations Form a Separate PCA Cluster

**Figure 4.**
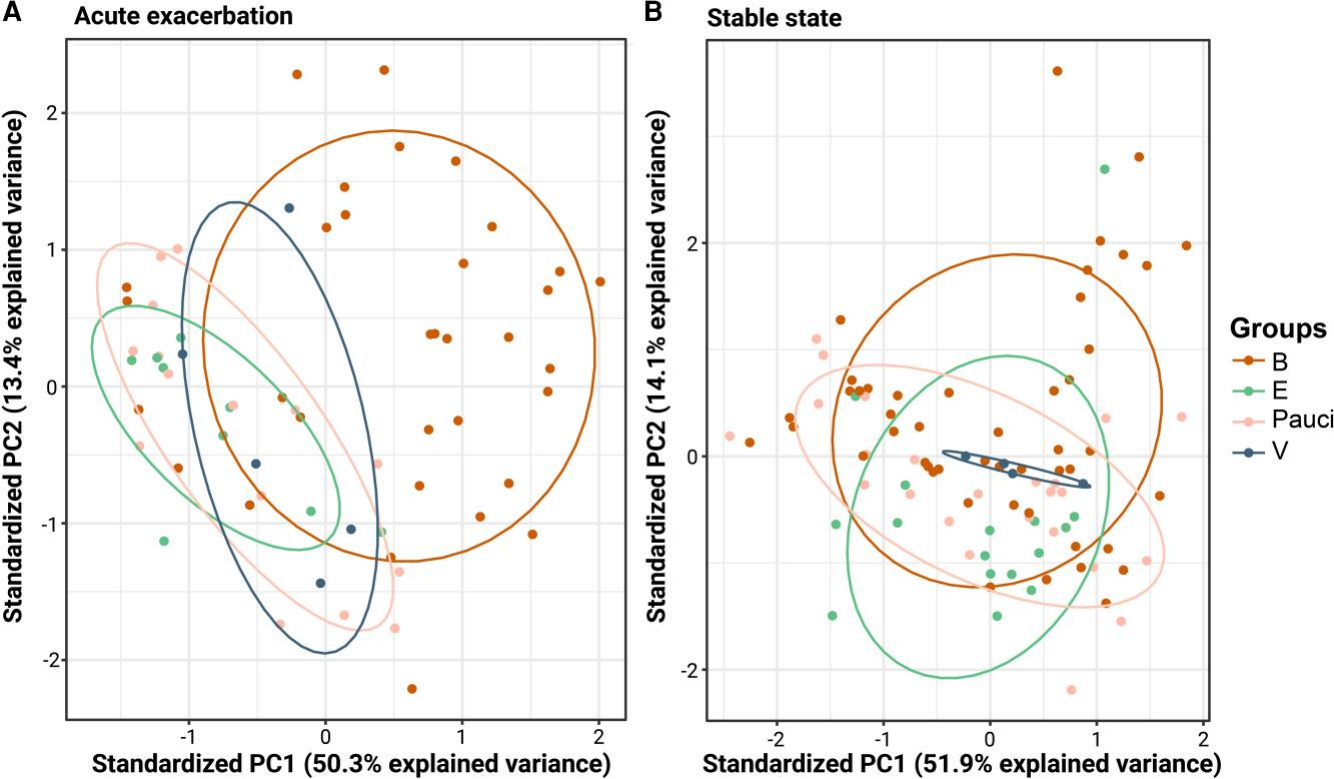
Principal component analysis (PCA) distinguishes a cluster of bacterial exacerbation state (EX) samples from other exacerbation types and disease states. PCA scatterplots, each dot represents 1 sample, color-coded by exacerbation type. *A*, PCA of 63 EX sputum samples, 1 per participant. *B*, PCA of 93 stable state samples, 1 per participant. Abbreviations: B, bacterial; E, eosinophilic; PC, principal component; V, viral.

PCAs identified the PCs that accounted for most variation between exacerbation types in the ST and EX datasets (Figure 4). The scatterplot of EX sputum samples revealed that 63.7% of the variance was explained by PC1 (50.3%) and PC2 (13.4%) and that bacteria-associated exacerbation formed a distinct cluster characterized by high PC1 and PC2 values (Figure 4*A*). In contrast, the clusters associated with V, E, and Pauci overlapped (Figure 4*A*).

The PCs were used to build a logistic model to predict bacteria-associated exacerbations:


y=βo+β1PC1+β2PC2+β3PC3

where y is 1 if the exacerbation is bacteria-associated or 0 if the exacerbation is not bacteria-associated (V, E, or Pauci). β1, β2, and β3 are the PCs’ respective coefficients, and βo is the intercept. To evaluate the model's performance, we randomly split the data into a training and a test set. Model parameters were evaluated on a balanced training set containing 70% of the data. Only PC1 and PC2 had a significant positive effect in predicting bacterial exacerbation. By applying the model to the independent test set, we measured an accuracy of 88%, specificity of 83%, and sensitivity of 90%.

The scatterplot of ST sputum samples revealed that 66.0% of the variance was explained by PC1 (51.9%) and PC2 (14.1%). None of the exacerbation type groups formed a distinct cluster (Figure 4*B*).

## DISCUSSION

This exploratory study showed the feasibility of monitoring multiple cytokines in sputum from COPD patients. We applied univariate and multivariate analyses to examine the expression of the cytokines, comparing disease states and exacerbation types. This way, we identified a specific cytokine signature that correlated with bacteria-associated exacerbations.

Increased levels of IL-17A, TNF-α, IL-1β, and IL-10 were detected in sputum during the EX compared to the ST visits. We should consider that COPD is a complex and multifactorial disease, where inflammation represents 1 of the factors involved in pathogenesis, and cytokines may play an important role in amplifying or regulating the inflammatory response. For instance, IL-1 is implicated in inflammation and may promote the production of other proinflammatory cytokines, although excessive production of IL-1 could contribute to lung tissue destruction. In this context, IL-1 inhibitors have been studied as a potential treatment for COPD [10]. Similarly, TNF-α is also a potent proinflammatory cytokine involved in the regulation of various immune responses. In COPD exacerbation, TNF is produced in excess and contributes to the recruitment and activation of immune cells, particularly neutrophils and macrophages, and induces the release of other inflammatory cytokines, amplifying the inflammatory response and contributing to tissue damage and airflow limitation [11, 12]. Finally, while the role of IL-17A in COPD is still being investigated, some studies suggest that IL-17A may contribute to chronic lung inflammation and worsening of symptoms [13]. The raised levels of the proinflammatory cytokines we detected in sputum during the EX compared to the ST visits corroborate the above considerations.

However, due to the low sensitivity, the model based on the cytokine data could not be used to differentiate the disease states. In a previous study, sputum IL-1β and TNF-α concentrations were also increased in EX versus ST. That study found TNFRI, TNFRII, IL-6, C-C motif chemokine ligand (CCL) 5, and CCL4 concentrations to be increased as well [6], but except for IL-6, these were not included in our 30-plex. In addition, none of their biomarkers differentiated disease states [6]. Interestingly, although IL-10 and IL-17A were included in their analyses, these were not increased in their EX samples. A smaller number of EX samples (n = 182) compared to our dataset (n = 235) may have contributed to this difference, as well as different exacerbation type definitions. While we excluded all samples with mixed types, they included mixed samples and categorized them based on the predominant exacerbation type [6]. In another study, TNF-α was also significantly different between ST and EX sputum samples, while IL-17A, IL-1β, and IL-10 were not measured [5].

When comparing exacerbation types, we found that bacteria-associated exacerbations had a clear proinflammatory cytokine signature based on high expression of IL-17A, TNF-α, IL-1β, and IL-1α, distinct from the other exacerbation types. These results confirm that bacterial exacerbations in COPD are commonly associated with increased production of proinflammatory cytokines. Also, in line with the cytokine signature is that IL-1 inhibitors have been studied as potential COPD treatment [10].

In Sethi and colleagues’ study of COPD patients with only bacterial exacerbations, TNF-α concentration was also significantly increased in EX sputum [4]. They detected significant increases of IL-8 and neutrophil elastase as well; however, these were not analyzed in our study. They did not analyze IL-17A, IL-1β, or IL-1α in their sputum samples [4]. In a small study focused on the lung microbiome, TNF-α was high in the cluster of (mainly EX) samples enriched for the bacterial pathogens *Pseudomonas* and *Haemophilus* compared to clusters enriched for other bacteria or mainly composed of ST samples. IL-17A was also analyzed in that study, but IL-1β and IL-1α were not [14]. In a study of sputum from ST patients, IL-1β, IL-10, and TNF-α were found to be increased in samples positive for *H influenzae* or *M catarrhalis*, 2 common pathogens in AECOPD, and bacterial loads for both pathogens were correlated with TNF-α [15].

In further analyses, we found that IL-1α, IL-1β, and TNF-α were the most suitable biomarkers for predicting bacteria-associated exacerbations. These results were confirmed in the multivariate modeling using PCA, where bacteria-associated exacerbations formed a cluster distinct from the other exacerbation types. A logistic model based on the PCs was able to determine whether an exacerbation was bacteria-associated with high accuracy, specificity, and sensitivity. This finding may provide a rapid, point-of-care assessment to distinguish bacteria-associated AECOPD from AECOPD due to other causes, which can be used in a routine clinical care setting. Being able to distinguish different causes of AECOPD quickly will improve timely treatment with appropriate medication and may thus improve patients’ recovery.

Previously, also in the AERIS cohort, it was found that increased eosinophils in the blood (≥2%) at ST predicted eosinophil-associated inflammation at EX visits [16]. Together with the biomarkers we identified for predicting bacteria-associated exacerbations, we can now distinguish 2 of 4 exacerbation types. The 4 exacerbation types likely represent discrete pathophysiological entities. The ability to identify bacteria-associated exacerbations and the convenience of performing the test on sputum rather than on a blood sample will likely affect the management of AECOPD, which commonly includes antibiotic treatment for all exacerbations [17], and may thus reduce unnecessary antibiotic use.

The evidence obtained through our study aligns with the fact that bacterial exacerbations in COPD are commonly associated with increased production of proinflammatory cytokines, including IL-1, TNF-α, IL-10, and IL-17A [6, 11, 13]. Indeed, bacterial exacerbations occur when a superimposed bacterial infection arises on the existing chronic inflammation and airway damage. The bacteria trigger a heightened immune response, leading to the release of various inflammatory mediators, including the cytokines mentioned above. On the other hand, viral exacerbations are triggered by viral respiratory infections that typically stimulate a different cytokine profile. The production of the type I interferons IFN-α and IFN-β (not measured in our study) is a hallmark of antiviral immune responses. However, in viral exacerbations, other cytokines, such as IL-6 and IL-8, may be involved, contributing to airway inflammation and symptoms [18, 19]. Finally, eosinophilic exacerbations, which are characterized by an increase in eosinophils in the airways, are often associated with an allergic or atopic component and the cytokine profile in eosinophilic exacerbations involves IL-5, which plays a central role in eosinophil recruitment and activation [20, 21]. Moreover, cytokines such as IL-4 and IL-13, which are associated with Th2-type immune responses, are also increased in eosinophilic exacerbations [21].

One limitation of our study was that while 936 samples were analyzed in total, only 235 of these (25.1%) were from EX visits, resulting in a relatively small sample size for the subsets of exacerbation types studied. This may have affected the possibility of detecting a significant difference between the groups, if present. Nonetheless, this is the largest number of EX samples in which biomarkers were analyzed, as the other studies referenced here included 177 [4], 148 [6], 36 [5], and 31 [14] EX sputum samples. Future studies should strive to verify our findings in larger cohorts. Furthermore, a second limitation lies in the fact that this is a single-center study performed in the United Kingdom, which may have limited ethnic diversity. Therefore, our findings must be replicated across multiple centers and ethnicities to determine whether they are universal before they can be implemented in clinical settings. A third limitation of our study is that although we analyzed many cytokines, some markers assessed in other studies were not analyzed. Therefore, our analyses need to be extended to include all markers that have been reported to differentiate between disease states or exacerbation types in various studies.

A strength of the study was using an electrochemiluminescence-based multiplex immunoassay that allowed for the simultaneous, sensitive, and accurate analysis of a large number of compounds, in this case 30 cytokines and chemokines, in a single small sample. The assay generates a large number of data points that can be investigated quickly to identify suitable biological markers.

In conclusion, the concentration of cytokines in sputum of AECOPD patients can predict with high confidence whether the exacerbation is associated with a bacterial infection. In particular, increased concentrations of IL-1α, IL-1β, and TNF-α indicate a bacteria-associated exacerbation, while in combinations these cytokines provide predictions with 97%–100% specificity. This finding provides a rapid, point-of-care assessment to distinguish bacteria-associated AECOPD from AECOPD due to other causes, which can be used in a routine clinical care setting. It can thus help avoid the unnecessary use of antibiotics and potentially improve exacerbation treatment and patients’ quality of life.

## Supplementary Data

Supplementary materials are available at *The Journal of Infectious Diseases* online (http://jid.oxfordjournals.org/). Supplementary materials consist of data provided by the author that are published to benefit the reader. The posted materials are not copyedited. The contents of all supplementary data are the sole responsibility of the authors. Questions or messages regarding errors should be addressed to the author.

## Supplementary Material

jiae232_Supplementary_Data

## Notes

***Acknowledgments.*** The authors thank Ashwani K. Arora (GSK) for his contribution to the study and his critical review of the manuscript; Ida Paciello (Toscana Life Sciences) for her contribution to the data acquisition; and the members of the AERIS and GSK study groups for their contribution to the study. The authors thank Business & Decision Life Sciences Medical Communication Service Center for editorial assistance and manuscript coordination, on behalf of GSK. Esther van de Vosse, on behalf of GSK, provided medical writing support. Meso Scale Discovery (MSD), SECTOR, V-plex, and Discovery Workbench are trademarks of Meso Scale Diagnostics, LLC.

***Author contributions.*** S. B., C. L., S. R., V. W., and S. R. P. contributed to the study design and methods. M. T., S. A., M. Ba., and M. Br. were responsible for data acquisition. M. T., S. B., M. S., F. S., M. Ba., C. L., S. R., M. C., T. M. A. W., M. Br., and S. R. P. performed data analysis and/or data interpretation. All authors read and edited the manuscript. All authors had full access to all the data, approved the final version of the manuscript, and had final responsibility for the decision to submit for publication.

***Data availability.*** Anonymized individual participant data and study documents can be requested for further research from www.clinicalstudydatarequest.com.

***Disclaimer.*** GlaxoSmithKline Biologicals SA was involved in all stages of study conduct, including analysis of the data. GlaxoSmithKline Biologicals SA also took charge of all costs associated with the development and publication of this manuscript.

***Financial support*.** This work (NCT01360398) was supported by GlaxoSmithKline Biologicals SA.
