# Supplementary material for: Cytokine Biomarkers of Exacerbations in Sputum From Patients With Chronic Obstructive Pulmonary Disease: A Prospective Cohort Study
Source: J Infect Dis. 2024 Jun 5;230(5):e1112–20. doi: 10.1093/infdis/jiae232 (PMC11566228; doi:10.1093/infdis/jiae232)
Supplement: jiae232_Supplementary_Data [file jiae232_supplementary_data.docx]

# Supplementary material

Supplementary Methods

Sputum sample processing - Biomarker testing

Up to 0.2 g of sputum were used for sample preparation for biomarker testing (Dithiothreitol [DTT], and phosphate-buffered saline [PBS] supernatant and pellet) and determination of the quality of the sputum sample by cytospin. Sputum sample was kept on ice until final processing for biomarker. Biomarker testing was performed within 2 hours after sputum collection. The number of secondary samples for biomarker testing depended on the amount of sputum collected. Figure below summarizes the process of sputum processing for biomarker testing.


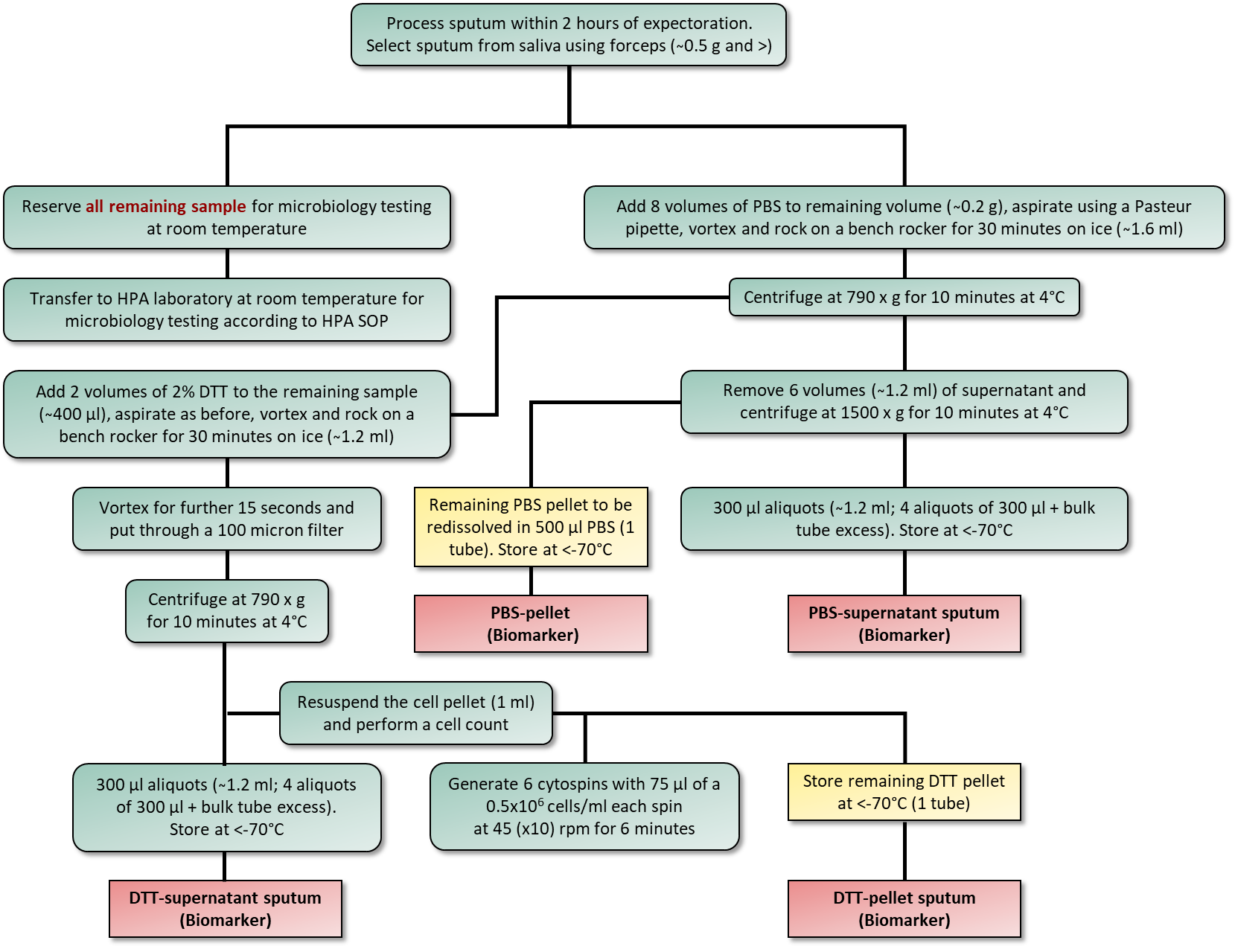


DTT, Dithiothreitol; HPA, Health Protection Agency (now Public Health England); PBS, phosphate-buffered saline; SOP, standard operating procedure.

Acceptance criteria

Plate

- Evaluation of Standard Curve estimating the Mean Relative Error (M.R.E).

M.R.E.% = Σ |R.E.%|/n

RE% is the relative error of each standard point and is calculated as the difference between the expected and observed values:

R.E.% =100* (V_observed_ – V_expected_) / V_expected_

n is the total number of standard points.

- The plate is valid if M.R.E.% ≤15%.
- Evaluation of Recovery Percentage for three Meso Scale Discovery (MSD) kit controls (High Medium and Low concentration).
- In order to accept the run, two out of three MSD controls must have a Percentage of Recovery between 70% and 130% for each cytokine.

Sample

- The concentration value is considered valid if it is within the quantification range lower limit of quantification (LLOQ) < Value < upper limit of quantification (ULOQ).
- A concentration values above the ULOQ or below the LLOQ cannot be considered as valid since precision and linearity in these regions were not assessed.
- The NaN (not a number) value is considered as not quantifiable cytokine, and no retest is required.

Spike and Recovery

To evaluate the impact of sputum matrix on analytes quantification, three samples with the lowest frequency of analytes above the LLOQ were selected.

The spike and recovery experiments were performed by adding a known amount of analyte to the sputum matrix and by calculating the % of Recovery of the analyte.

- If the % of Recovery is between 70% and 130% the impact of matrix is considered negligible.
- If the % of Recovery is < 70% or >130% the impact of matrix is considered relevant when 2 out of 3 controls are outside the defined range.

Spike and Recovery experiment showed that the quantification of 27 out of 29 analytes was not impacted by the sputum matrix. The matrix impact on IL-2 and TNF-α quantification was minor. IL-8 quantification was only performed through IL-8HA.

**Supplementary Table S1 Viruses tested in the sputum samples**

| **Virus** |
| --- |
| Enterovirus |
| Rhinovirus |
| Adenovirus |
| Influenza viruses A |
| Influenza virus B |
| Human coronaviruses 229E |
| Human coronaviruses HKU1 |
| Human coronaviruses NL63 |
| Human coronaviruses OC43 |
| Human metapneumovirus |
| Human bocavirus |
| Parainfluenza virus 1 |
| Parainfluenza virus 2 |
| Parainfluenza virus 3 |
| Parainfluenza virus 4 |
| Human respiratory virus |
| Respiratory syncytial virus |

**Supplementary Table S2 Cytokines and chemokines in the V-plex 30-plex panel kit**

| **Proinflammatory panel** | **Cytokine panel** | **Chemokine panel** |
| --- | --- | --- |
| Interferon (IFN)-γ | Granulocyte-macrophage colony-stimulating factor (GM-CSF)* | Eotaxin** |
| IL (Interleukin)-1β | IL-1α | Macrophage inflammatory protein (MIP)-1β** |
| IL-2 | IL-5 | Eotaxin-3** |
| IL-4 | IL-7 | Thymus and activation regulated chemokine (TARC)** |
| IL-6 | IL-12/IL-23p40** | IFN-γ-induced protein (IP)-10** |
| IL-8* | IL-15 | MIP-1α |
| IL-10 | IL-16 | IL-8HA** |
| IL-12p70 | IL-17A | Monocyte chemoattractant protein (MCP)-1 |
| IL-13 | TNF-β* | Macrophage-derived chemokine (MDC)** |
| Tumor necrosis factor (TNF)-α | Vascular endothelial growth factor A (VEGF-A)* | MCP-4** |

The V-plex 30-plex panel kit consists of three independent 10-plex panels: the Proinflammatory panel, the Cytokine panel, and the Chemokine panel.

*Cytokine excluded as <20% of the values were between the LLOQ and ULOQ.

**Cytokine excluded as ≥8% of samples did not generate valid results.

LLOQ, lower limit of quantification; ULOQ, upper limit of quantification.

## Supplementary Table S3 Combined contribution of IL-1α, IL-1β, and TNF-α as biomarkers for bacteria-associated exacerbations

| **Biomarker combination** | **Sensitivity*** | **Specificity**** | **Accuracy** |
| --- | --- | --- | --- |
| IL-1α + IL-1β | 70% | 97% | 83% |
| IL-1β + TNF-α | 70% | 100% | 84% |
| IL-1α + TNF-α | 79% | 97% | 87% |
| IL-1α + IL-1β + TNF-α | 70% | 100% | 84% |

*Percentage of bacterial exacerbations correctly predicted. **Percentage of no-bacterial exacerbation correctly predicted.

IL, interleukin; TNF, tumor necrosis factor.

## Supplementary Figure S1 Distribution of the raw data from the cytokine measurements


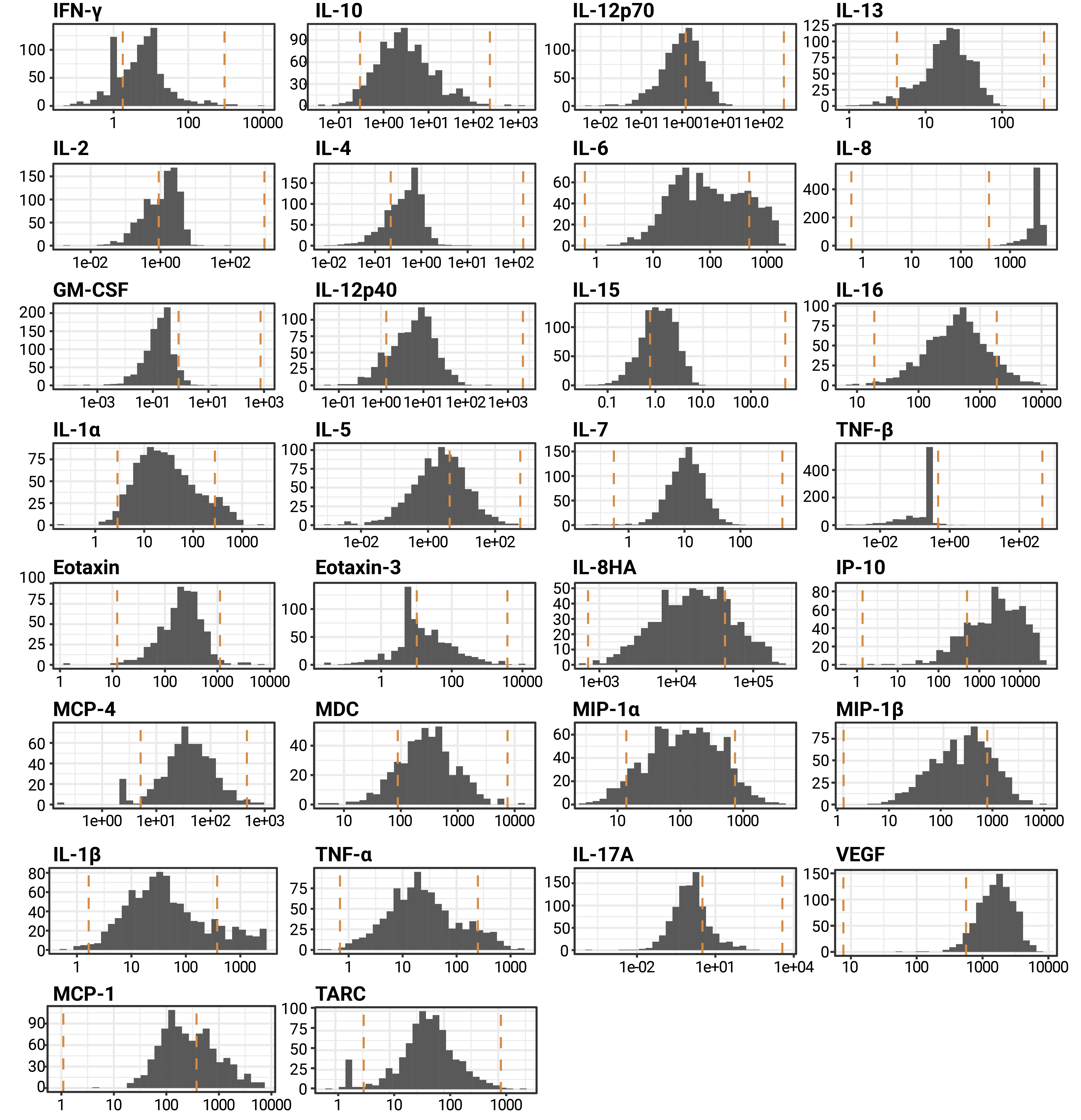


In each panel, the horizontal axis indicates the concentration (pg/ml) of a given cytokine (on a log10 scale) and the y-axis is the count (the number of observations per interval of concentration). Red lines represent the lower limit and the upper limit of the quantification range. GM-CSF, granulocyte-macrophage colony-stimulating factor; IFN, interferon; IL, interleukin; IP, IFN-γ-induced protein; MCP, monocyte chemoattractant protein; MDC, macrophage-derived chemokine; MIP, macrophage inflammatory protein; TARC, thymus and activation regulated chemokine; TNF, tumor necrosis factor; VEGF, vascular endothelial growth factor.

## Supplementary Figure S2 ROC curves for prediction of bacteria-associated exacerbation


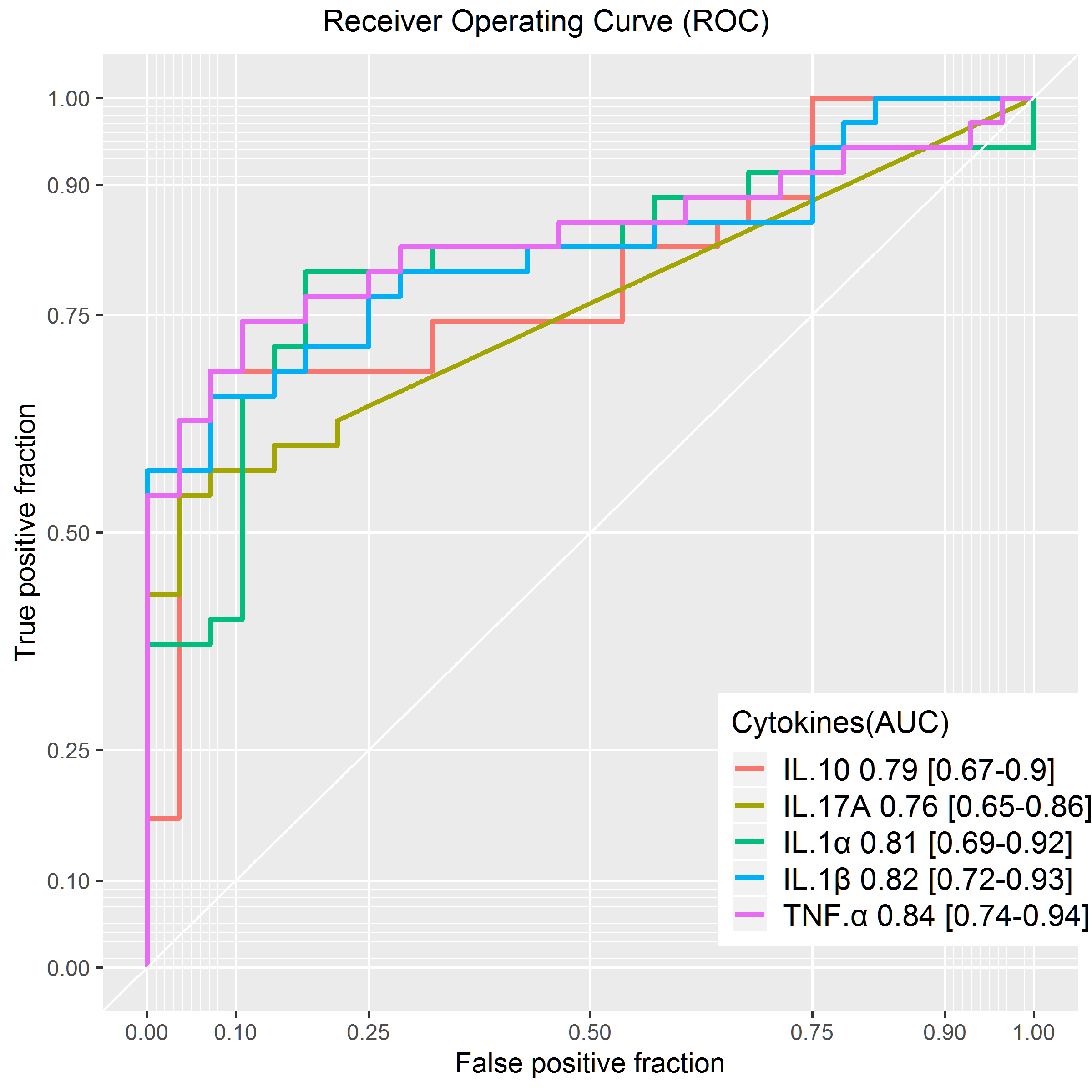


Depicted are the ROC curves of cytokines for which the AUC was >0.75. The AUC and 95% CI are shown in the legend.

AUC, area under the curve; CI, confidence interval; IL, interleukin; ROC, receiver operating characteristic; TNF, tumor necrosis factor.
